# Multidisciplinary Management of Esophageal Perforation and Septic Shock Following Peroral Endoscopic Myotomy in an Elderly Patient: A Case Report

**DOI:** 10.7759/cureus.107233

**Published:** 2026-04-17

**Authors:** Kamilla Beisenova, Victoria Stoffel, Huzaifa Shakir

**Affiliations:** 1 Department of Medicine, University of New England College of Medicine, Biddeford, USA; 2 Department of Surgery, Rutgers New Jersey Medical School, Newark, USA; 3 Department of Cardiothoracic Surgery, Rutgers New Jersey Medical School, Newark, USA

**Keywords:** esophageal necrosis, esophageal perforation, esophageal stent, esophagectomy, septic shock

## Abstract

Esophageal stents are indicated for strictures, leaks, and perforations, but contraindications include uncontained perforations, uncontrolled sepsis, and necrotic tissue. We report a case of a 73-year-old female with an esophageal perforation after peroral endoscopic myotomy who underwent chest tube placement, video-assisted thoracoscopic surgery with attempted repair, and placement of two overlapping esophageal stents extending into the stomach. Despite stenting, she further decompensated and developed esophageal necrosis requiring esophagectomy, removal of necrotic tissue, creation of a spit fistula, and retrieval of both stents. Although within the allowable length, the stents were placed in necrotic tissue and extended into the stomach, resulting in further complications that could have been avoided with early surgical intervention. This case underscores that stenting, especially in excessive length, in necrotic tissue, can worsen necrosis and complicate surgery. In such cases, early esophagectomy may be the more definitive option in these scenarios.

## Introduction

Peroral endoscopic myotomy (POEM) is a well-established, minimally invasive procedure used to treat achalasia and other motility disorders [1]. Although POEM is generally safe, it carries multiple risks, including the rare but potentially life-threatening complication of esophageal perforation reported in approximately 0.5-3% of cases [2]. Esophageal perforation requires prompt recognition, as delay in treatment may cause complications such as mediastinitis and sepsis. Treatment strategies vary based on the size and location of the perforation, clinical status of the patient, and the degree of contamination. Esophageal perforations are often managed with endoscopic esophageal stenting, typically performed by the gastrointestinal team. However, esophageal stenting is also associated with complications, including bleeding, stent migration, and pressure necrosis [3]. We present a case of a 73-year-old female who developed septic shock following an esophageal perforation after POEM. Despite initial management with chest tube drainage, endoscopic suturing, and stent placement, her course was complicated by persistent leak, progressive necrosis, and eventual need for esophagectomy. This case underscores the challenges of managing esophageal perforation in compromised tissue and highlights the importance of appropriate stent selection, recognition of contraindications, and timely multidisciplinary intervention.

## Case presentation

**Figure 1:**
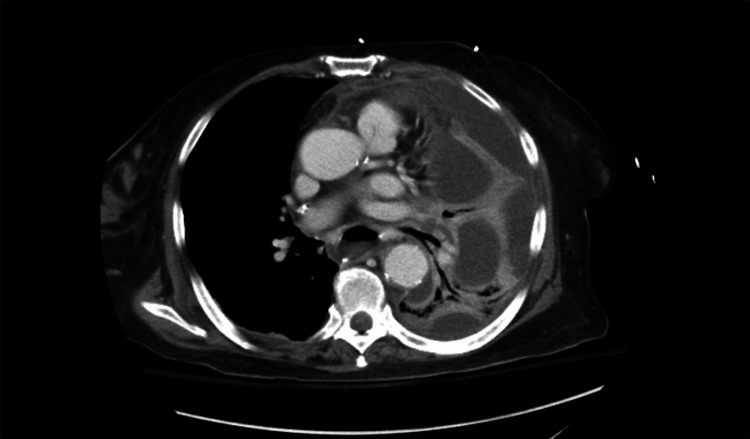
Chest computed tomography demonstrating a left-sided pleural effusion (arrow) with associated findings concerning for esophageal perforation, including adjacent mediastinal contamination. These findings correlate with the patient’s clinical presentation of chest pain and progression to septic shock.

A 73-year-old female presented to the hospital several weeks after an attempted peroral endoscopic myotomy performed by the gastrointestinal service for achalasia. She complained of chest pain and was found to have an esophageal perforation with contamination of the left chest, which led to septic shock (Figure 1).

**Figure 2:**
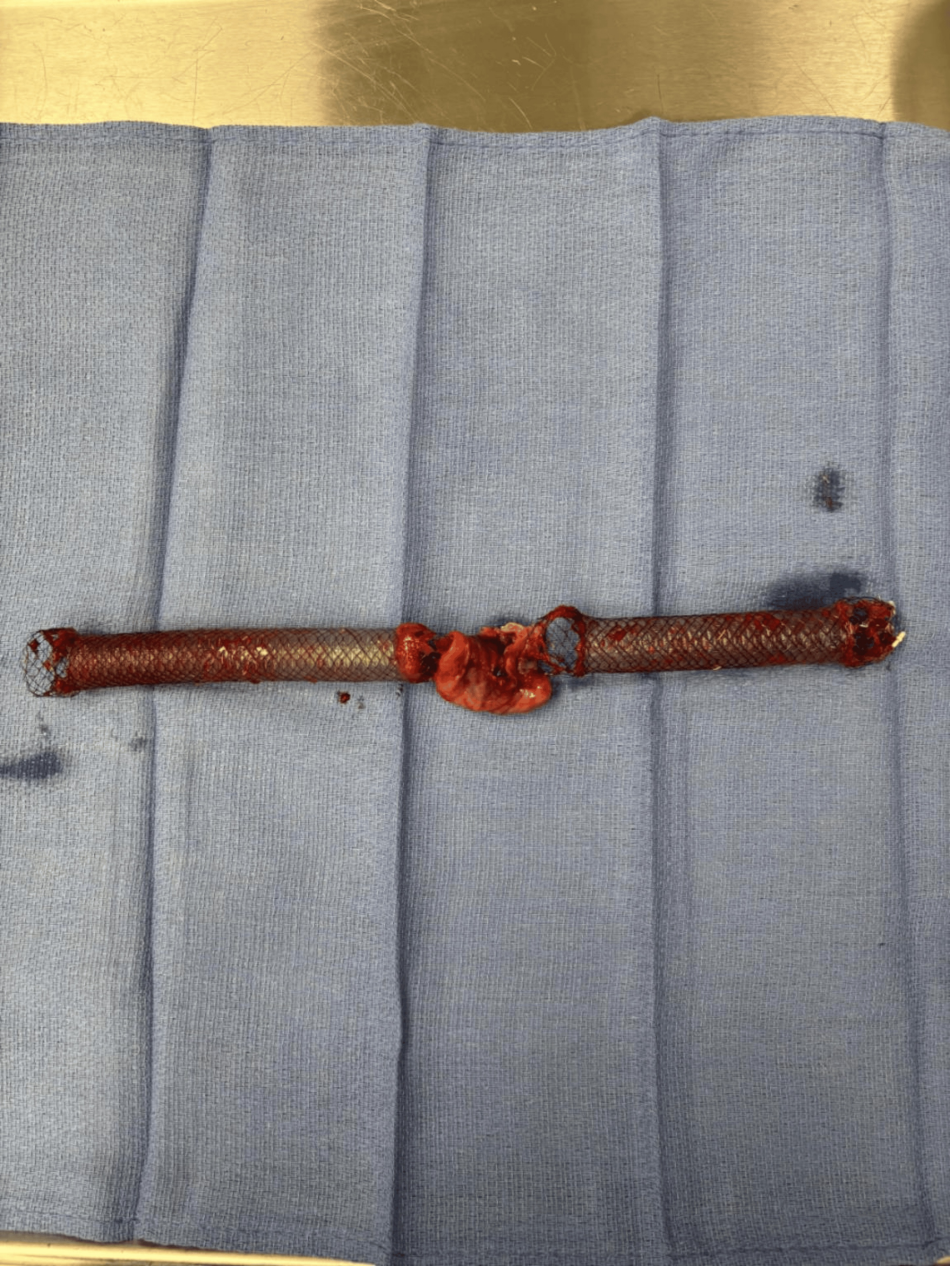
Excised esophageal stents measuring 15 cm and 12 cm following esophagectomy. The use of overlapping stents resulted in extensive coverage of the esophagus, with distal extension into the stomach, which may have contributed to ongoing tissue injury and complications in the setting of necrotic esophageal tissue.

Her medical history was notable for adenocarcinoma of the right lung, which was resected in 2021, a cerebral vascular accident in 2019, hypertension, hyperlipidemia, chronic kidney disease, depression, and achalasia. She was initially admitted to the medical intensive care unit, where she underwent left chest tube placement and esophagogastroduodenoscopy (EGD) with endoscopic suture and stent post-procedure day (PPD) 0. Despite this, she continued to have a persistent leak and decompensated in the medical intensive care unit. She was subsequently admitted to the cardiothoracic intensive care unit. On PPD 5, she underwent a left video-assisted thoracoscopic surgery (VATS) with washout of the left chest, decortication, mediastinal exploration, and re-closure of the esophageal perforation. Following the VATS procedure, the gastrointestinal team introduced an EGD scope while the patient was still intubated. The previous esophageal stent was pulled out, and the site of esophageal perforation was visualized. The sutures from the previous endoscopy had given way due to necrotic tissue. They were removed. Two attempts were made to place new sutures and close the defect; however, due to the necrotic tissue, approximation of the perforation was unsuccessful. To prevent leakage, the decision was made to place an esophageal stent across the perforation. Two overlapping stents, measuring 12 cm and 15 cm each, were placed across the perforation, with a portion of the stent extending into the stomach (Figure 2).

The stents were then sutured in place. Postoperatively, she was found to have a persistently elevated lactate and was unable to be weaned off pressors, which prompted a return to the operating room on PPD 7 for more extensive intervention. She underwent esophagectomy, removal of necrotic tissue, creation of a spit fistula, and placement of Moss and Malecot feeding tubes into the mediastinum and stomach. During the dissection, the esophagus was found to be necrotic and adhered to the surrounding tissues. The first esophageal stent became visible and was removed along with part of the proximal stomach. The stomach was transected proximal to the left gastric artery. As dissection continued circumferentially, the entire esophagus was noted to be nonviable. Both esophageal stents that had been in place were retrieved during this process. Because normal anatomy could not be clearly identified, the remaining stomach was oversewn, and decompression and feeding access were established using a Moss tube for drainage, and a distal feeding tube was advanced into the duodenum. Intraoperatively, she developed acute hypotension and tachycardia, requiring a right-sided chest tube for concern of pneumothorax. She was started on norepinephrine at 10 µg/min postoperatively and remained intubated and sedated. Postoperatively, she was managed in the surgical intensive care unit with supportive care for acute respiratory failure, which included ventilation on AC/VC (assist control/volume control), chest tube suctioning, and antibiotic therapy. She also received nutritional support via feeding tube, had renal monitoring for chronic kidney disease/acute kidney injury, and was treated for stress hyperglycemia. The patient’s intensive care unit (ICU) course included multidisciplinary support from the surgery, critical care, and infectious disease teams. She remained in the ICU under close monitoring, with follow-up focused on weaning sedation, managing infection, and nutritional support planning. Four weeks later, she was discharged to rehabilitation with a tracheostomy and a speaking valve.

Certain clinical details, including precise timing of events, initial vital signs, and select diagnostic parameters, were not consistently documented in the medical record and therefore could not be included. This represents a limitation of the case report.

## Discussion

Esophageal stents are indicated in cases of malignant esophageal strictures, benign esophageal strictures, tracheoesophageal fistulas, esophageal leaks and perforations, palliative management, anastomotic leaks, and prevention of re-leak in esophageal perforation management [4]. Contraindications to esophageal stents include uncontained perforations, uncontrolled sepsis, esophageal necrosis, severe coagulopathy, extremely tortuous or severely narrowed esophagus, and proximity to the upper esophageal sphincter. Esophageal stents typically range in diameters of 16-20 mm and lengths of 6-15 cm. Having a length that excessively overlaps normal mucosa is considered to be beyond the appropriate recommended sizing.

Current guidelines from the European Society of Gastrointestinal Endoscopy (ESGE) and the American Society for Gastrointestinal Endoscopy (ASGE) support the use of self-expandable metal stents (SEMS) for benign esophageal perforations, leaks, and fistulas [5,6]. For benign perforations, early diagnosis and intervention (within 24 hours) are critical predictors of clinical success [5]. Delayed recognition beyond 24 hours has been associated with increased risk of morbidity and mortality, largely due to the progression of contamination and sepsis, which further complicates endoscopic and surgical management. ESGE notes that while no specific stent type is universally recommended, SEMS generally show higher technical success and clinical efficacy compared to self-expandable plastic stents (SEPS) for these indications [5]. ASGE reports that SEMS treatment for esophageal perforations is associated with significantly better short-term outcomes than surgery, including faster resumption of oral diet and shorter hospital stays [6]. The success of stenting is influenced by the location of the injury, the size of the orifice, and inflammatory markers such as C-reactive protein (CRP) [5]. The size of the perforation is a critical factor in the determination of treatment strategy. Larger defects may pose the difficulty of achieving an adequate seal and thus have a higher risk of persistent leakage. The size of the esophageal perforation was not documented in the operative report or elsewhere in the medical record. As such, this information could not be included and represents a limitation of this case, particularly given its relevance in guiding management decisions and prognostication.

Previous studies have looked into the complications regarding excessive stent length, which causes unnecessary coverage of healthy tissue [7]. Complications include pressure necrosis, ulceration, and perforation of the esophageal wall [8]. According to the American Gastroenterological Association, an esophageal stent should extend approximately 2 cm beyond the proximal and distal margins of the stricture [9]. Careful selection of the stent length is essential to reduce serious complications.

In this case, the presence of necrotic tissue that was found after the original esophageal stent was placed posed an immediate contraindication to placing another stent. Although it was noted that the esophageal defect was initially closed with sutures, these could not hold because of the necrotic tissue. Hence, the decision to place a stent can be justified as an alternative attempt to close the perforation and prevent further leakage. It is important to point out that one of the two new stents extended into the stomach. This raises concern, as extending a stent too far into viable tissue, particularly into the gastric area, can cause additional complications.

On return to the operating room, more necrotic tissue was discovered. This suggests that the previously stented esophageal tissue was already necrotic and continued to deteriorate. Again, this reinforces the idea that an esophageal stent may have been contraindicated from the outset, as there was no viable esophageal tissue in which to anchor the stent. With the advanced necrosis, the stent was not effective and contributed to adherence of the necrotic esophagus to surrounding tissues. Furthermore, the stomach had to be transected in order to remove one of the two stents. During that surgery, the entire esophagus was noted to be non-viable.

High-risk perforations, such as those involving necrotic tissue or sepsis, require careful multidisciplinary evaluation as stenting can sometimes exacerbate the condition. Following ESGE and ASGE guidelines, stenting is generally contraindicated in the presence of active, uncontrolled sepsis or necrotic tissue [5]. Some surgical teams view esophageal stenting as a relative contraindication because the presence of a stent may compromise the ability to perform a primary surgical repair later [5]. In complex cases, using stents of excessive length or allowing them to extend into the stomach (to bridge a low perforation) significantly increases the risk of migration, reflux, and further tissue damage [5]. In scenarios where the esophagus is non-viable or the patient fails to stabilize after stenting, early esophagectomy may be the more definitive and necessary option. However, this decision needs to be made on an individualized basis, hinging on the patient's clinical status, extent of the disease, and multidisciplinary evaluation.

Taking into account that the average esophageal length in an adult woman is approximately 23-24 cm (measured from the upper esophageal sphincter to the gastroesophageal junction), the two stents, measuring 15 cm and 12 cm, had a combined length of 27 cm [10]. Although the stents overlapped and thus reduced the effective total length, the combined measurements came close to covering nearly the entire esophagus. Current data on esophageal stenting recommend that the stent extend at least 2 cm beyond the proximal and distal margins of the perforation. In this case, the stents would not have had sufficient room to extend into normal viable tissue, as there was little to no viable esophagus remaining. Additionally, extension of the stent into the stomach increases the risk of migration, reflux, and aspiration, especially if the stent crosses the gastroesophageal junction and an antireflux valve is not in place [6]. Even with acid suppression and antireflux medications, complications in this case are frequent.

Although both stents used during the second procedure were technically within the maximum allowable esophageal stent length, the extent of necrosis raises the question of whether esophageal stenting was appropriate in the first place, and whether an esophagectomy would have been the more definitive option at the time of the initial operation. Placing stents in compromised tissue is said to have complications of pain, migration, recurrent dysphagia, ulceration, and perforation [11].

This case illustrates several key factors that influenced the patient’s clinical course, including the presence of necrotic esophageal tissue, persistent sepsis, and the use of overlapping stents with distal extension into the stomach. These findings highlight the importance of careful patient selection, the full clinical picture, and procedural planning when considering esophageal stenting. In particular, the lack of viable tissue likely limited the effectiveness of endoscopic interventions and contributed to ongoing deterioration, which ultimately required surgical management.

## Conclusions

In summary, this case highlights the importance of recognizing appropriate indications and limitations of esophageal stent placement in the management of esophageal perforation. Excessive stent length, placement into the stomach, as well as placement in necrotic tissue, may increase the risk of complications and consequently risk complicating further surgical intervention. While esophageal stenting remains an important therapeutic option, careful patient selection and multidisciplinary evaluation are essential. In complex cases involving extensive tissue compromise or clinical deterioration, early surgical intervention, including esophagectomy, may be considered; however, management should be individualized based on patient condition and intraoperative findings. This case highlights the importance of adhering to guideline indications for stenting and the need for multidisciplinary discussion and prompt decision-making in managing complex esophageal perforations.
